# Correction: *Drosophila americana* Diapausing Females Show Features Typical of Young Flies

**DOI:** 10.1371/journal.pone.0142009

**Published:** 2015-11-06

**Authors:** The PLOS ONE Staff

## Notice of Republication

This article was republished on October 16, 2015, to correct the presentation of the abstract, which was incorrectly tagged in the article XML. The publisher apologizes for the error. The correct abstract is:

**Abstract**

Diapause is a period of arrested development which is controlled physiologically, preprogrammed environmentally and characterized by metabolic depression that can occur during any stage of insect development. Nevertheless, in the genus *Drosophila*, diapause is almost always associated with the cessation of ovarian development and reproductive activity in adult females. In this work, we show that, in *D*. *americana* (a temperate species of the *virilis* group), diapause is a genetically determined delay in ovarian development that is triggered by temperature and/or photoperiod. Moreover, we show that in this species diapause incidence increases with latitude, ranging from 13% in the southernmost to 91% in the northernmost range of the distribution. When exposed to diapause inducing conditions, both diapausing and non-diapausing females show a 10% increase in lifespan, that is further increased by 18.6% in diapausing females, although senescence is far from being negligible. *ActinD1* expression levels suggest that diapausing females are biologically much younger than their chronological age, and that the fly as a whole, rather than the ovarian development alone, which is phenotypically more evident, is delayed by diapause. Therefore, diapause candidate genes that show expression levels that are compatible with flies younger than their chronological age may not necessarily play a role in reproductive diapause and in adaptation to seasonally varying environmental conditions.

Please download this article again to view the correct version. The originally published, uncorrected article and the republished, corrected article are provided here for reference.

## Supporting Information

S1 File Originally published, uncorrected article. (PDF)

S2 File Republished, corrected article. (PDF)
