# Supplementary material for: Correction: Drosophila americana Diapausing Females Show Features Typical of Young Flies
Source: PLoS One. 2015 Nov 6;10(11):e0142009. doi: 10.1371/journal.pone.0142009 (PMC4636155; doi:10.1371/journal.pone.0142009)
Supplement: S1 File — (PDF) [file pone.0142009.s001.pdf]

RESEARCH ARTICLE

Drosophila americana Diapausing Females Show Features Typical of Young Flies

Micael Reis[1,2], **Felipe B. Valer**[2,3], **Cristina P. Vieira**[1,2], **Jorge Vieira**[1,2]*

1 Instituto de Investigação e Inovação em Saúde, Universidade do Porto, Porto, Portugal, **2** Instituto de Biologia Molecular e Celular (IBMC), Universidade do Porto, Porto, Portugal, **3** Instituto de Biologia, Universidade Federal de Pelotas—UFPel, Pelotas, Rio Grande do Sul, Brazil

* jbvieira@ibmc.up.pt

Diapause is a period of arrested development which is controlled physiologically, preprogrammed environmentally and characterized by metabolic depression that can occur during any stage of insect development. Nevertheless, in the genus *Drosophila*, diapause is almost always associated with the cessation of ovarian development and reproductive activity in adult females. In this work, we show that, in *D. americana* (a temperate species of the *virilis* group), diapause is a genetically determined delay in ovarian development that is triggered by temperature and/or photoperiod. Moreover, we show that in this species diapause incidence increases with latitude, ranging from 13% in the southernmost to 91% in the northernmost range of the distribution. When exposed to diapause inducing conditions, both diapausing and non-diapausing females show a 10% increase in lifespan, that is further increased by 18.6% in diapausing females, although senescence is far from being negligible. *ActinD1* expression levels suggest that diapausing females are biologically much younger than their chronological age, and that the fly as a whole, rather than the ovarian development alone, which is phenotypically more evident, is delayed by diapause. Therefore, diapause candidate genes that show expression levels that are compatible with flies younger than their chronological age may not necessarily play a role in reproductive diapause and in adaptation to seasonally varying environmental conditions.

CrossMark
click for updates

OPEN ACCESS

Citation: Reis M, Valer FB, Vieira CP, Vieira J (2015) *Drosophila americana* Diapausing Females Show Features Typical of Young Flies. PLoS ONE 10(9): e0138758. doi:10.1371/journal.pone.0138758

Editor: Patrick O'Grady, University of California, Berkeley, UNITED STATES

Received: January 20, 2014

Accepted: September 3, 2015

Published: September 23, 2015

Copyright: © 2015 Reis et al. This is an open access article distributed under the terms of the Creative Commons Attribution License, which permits unrestricted use, distribution, and reproduction in any medium, provided the original author and source are credited.

Funding: This work was financially supported by national funds through FCT - Fundação para a Ciência e a Tecnologia/MEC - Ministério da Educação e Ciência, and when applicable, co-funded by FEDER funds within the partnership agreement PT2020 related with the research unit number 4293. Micael Reis was funded by a PhD grant attributed by FCT with the reference SFRH/BD/61142/2009. The funders had no role in study design, data collection and analysis, decision to publish, or preparation of the manuscript.

Competing Interests: The authors have declared that no competing interests exist.

Introduction

Diapause was initially described by Henneguy [1] as a stage of delay in ontogenetic development. Currently, it is defined as a period of arrested development which is controlled physiologically, preprogrammed environmentally and characterized by metabolic depression that can occur during any stage of insect development [2], and thus, different from quiescence, which implies immediate direct response to a limiting factor, such as cessation of development due to temperature, and can be quickly reversed to normal conditions [3]. Depending on the insect species, diapause can occur at different stages of the life cycle, from embryo to adults [4] and be triggered by diverse environmental cues, including temperature, humidity or photoperiod [5].

In *Drosophila*, reproductive diapause is observed in adult females and is defined as the cessation of ovarian development and reproductive activity at an immature stage [6–10]. In the northern species from the *Drosophila* subgenus, photoperiod is the most reliable diapause inducing cue, although low temperatures seem to be also important [11, 12]. Moreover, in species from the *D. auraria* complex (*Sophophora* subgenus), photoperiodic changes are also important for the setting of diapause [10, 13]. Nevertheless, in *D. melanogaster* (*Sophophora* subgenus), the relative importance of photoperiod and low temperatures (such as 11°C) is not clear (e.g. [14, 15]). In *D. montana* (*Drosophila* subgenus), females must experience three or

more short day cycles during the sensitive period (while the ovaries are still immature) in order to diapause [16]. In this species, the sensitive period for diapause induction is shorter at higher temperatures (about 8 days after eclosion at 16°C and 4–5 days after eclosion at 19°C), since it is limited by the faster development rate of the ovaries at these temperatures [16].

At high latitudes, where the unfavorable season is longer, *Drosophila* females are expected to show a higher propensity to diapause than at low latitudes, where the unfavorable season is shorter. Compatible with this view, latitudinal gradients for diapause incidence have been reported for species from different geographical locations, such as, European populations of *D. littoralis* [6] and *D. montana* [9, 17], Japanese populations of *D. lacertosa* [7] and North American populations of *D. melanogaster* [8, 18]. Besides the varying latitudinal incidence, diapause also cycles temporally in *D. melanogaster* [19]. Life-history trade-offs, associated with variation for the diapause phenotype, likely maintain the observed gradients [8, 9, 20, 21].

Juvenile hormones, ecdysteroids, and the neuropeptides that govern juvenile hormone and ecdysteroid synthesis, as well as genes from the *insulin* signaling pathway and circadian clock genes have been shown to be important components of the regulatory pathway leading to the diapause phenotype [10, 22–26], and in *D. melanogaster*, allele gradients that correlate with diapause incidence have been reported for *timeless* (a circadian clock gene) in European populations [27, 28] and for *couch potato* (a gene that may make the link between the *insulin* signaling pathway and the hormonal control of diapause) in North American populations [18, 29]. Nevertheless, the *couch potato* variant that shows the strongest association with diapause incidence in North American populations is not correlated with diapause incidence in Australian populations [30].

The slower metabolism, as well as the increase in cold and starvation resistance that is characteristic of diapausing insects represents an advantage to cope with the long cyclic unfavorable climate conditions [2, 31–35]. Moreover, *D. melanogaster* females that have been in diapause for periods of time as long as nine weeks have the same post-diapausing lifespan (LS) as new born flies reared at 25°C [15, 36]. The reasons for such LS extension are not well understood, but may be related to the slower metabolism. It has been recently suggested that the rate of senescence of *D. melanogaster* female intestines is slow down under diapause inducing conditions [37]. Therefore, it is conceivable that the development of the female as a whole, rather than the ovarian development alone (which is phenotypically more evident; [6–10]), is delayed by diapause. This would imply that diapausing females are biologically much younger than their chronological age.

In this work, we show that, in *D. americana* (a temperate species of the *virilis* group), diapause is a genetically determined delay in ovarian development that is triggered by temperature and/or photoperiod. This species belongs to an old lineage of temperate species [38, 39], and thus, the ability to diapause has been very relevant during its evolutionary history. Diapause incidence increases with latitude, ranging from 13% in the southernmost range of the distribution to 91% in the northernmost range of the distribution. When exposed to diapause inducing conditions, both diapausing and non-diapausing females show a 10% increase in lifespan, that is further increased by 18.6% in diapausing females. Thus, senescence is far from being negligible. *ActinD1* expression levels suggest that diapausing females are biologically much younger than their chronological age. Interestingly, the development of the fly as a whole, rather than the ovarian development alone, which is phenotypically more evident, is delayed by diapause.

Materials and Methods

Fly strains

In order to identify strains showing a clear diapausing and non-diapausing phenotype, 39 *D. americana* strains from north, center and south of United States of America were assayed.

From the north of the distribution, isofemale strains (O30, O31, O33, O34, O35, O37, O39, O40, O43, O45, O50, O53, O57, O62 and O67) were established with flies collected at the end of July 2008, in Fremont (Nebraska). From the center of the distribution, isofemale strains were established with flies collected at the end of July and beginning of August 2004 from Howell Island, Missouri (HI14, HI18, HI23, HI25 and HI29); and from Lake Wappapelo, Missouri (W11, W25, W26, W29, W33, W36, W37 and W46). Moreover, from the south of the distribution, isofemale strains were established with flies collected in June 2005 in Corney Bayou, Louisiana (CB05.08, CB05.20, CB05.22 and CB05.24), at the end of May in Cat Island, Louisiana (CI05.20, CI05.28 and CI05.30), and during the summer of 2010 at Pearl River, Mississippi (RB10.20 and RB10.22) by Bryant McAllister (Iowa University, Iowa, USA), who kindly sent us the strains. Lastly, strains SF12 and SF14 were established with flies collected at the end of July 2010 at Saint Francisville, Louisiana.

Reproductive diapause phenotyping

For each strain described above, about 20 *D. americana* virgin female flies were collected under brief CO_2 anesthesia after eclosion at 25°C under 12h of light and 12h of dark cycles (12L:12D). Then, they were kept in bottles with culture medium in a climate chamber at 11°C under 10h of light and 14h of dark cycles (10L:14D) for 28 days (diapause inducing conditions) in the absence of males. Moreover, about 20 virgin female flies were collected and reared in bottles with culture medium under non-diapause inducing conditions (12L:12D at 25°C) for 28 days in order to check for complete oocytes development. When flies were 28 days old, all virgin females from each strain were individually examined for the developmental stage reached by the oocytes. Each female was anesthetized in CO_2 and dissected in a drop of 50 μl of PBS (Phosphate Buffered Saline) using jeweler's forceps to reveal the ovaries. Oocytes were found at various stages of development in the ovaries of each fly. The most advanced oocyte present in each female was used to categorize the fly according to the stages of ovary development described in [40]. Reproductive diapause is considered if ovarian development in all oocytes is arrested prior to vitellogenesis (the most advanced oocyte was $\leq$ stage seven). Evidence for virgin females showing immature ovaries (all the oocytes arrested prior to vitellogenesis) after 28 days under non-diapause inducing conditions was obtained for strains O45, W33 and W36. These strains were excluded from the analysis, since, when reared under non-diapause inducing conditions they already show a phenotype that is indistinguishable from the diapause phenotype. The sums of diapausing and non-diapausing females for each population were used to address whether the differences in diapause incidence between populations from the same geographical location are statistically significant, using two-by-two contingency tables and Fisher's exact test, implemented in SPSS Statistics 22.0 (IBM, New York, USA).

In order to elucidate the diapause phenotype, we decided to take pictures of ovaries of flies reared under non-diapause inducing conditions and ovaries of flies reared under diapause inducing conditions showing either diapausing or non-diapausing phenotypes. The flies were dissected as mentioned above and the pictures were taken using a stereomicroscope Nikon ZMS 1500 H with a magnification of 11.5X to include all three pairs of ovaries, and larger magnifications to get a closer look of ovaries of non-diapausing (60X) and diapausing females (112.5X). The resulting JPG files were saved with a resolution of 1600x1200 pixels. Then, the area of the different ovaries was measured by counting the number of pixels corresponding to these structures from the photo which captured all three types of ovaries (see Results) by using ImageJ (http://imagej.nih.gov/ij/, [41]). A scale was photographed also, under the same conditions, to allow for the conversion between number of pixels and μm^2. In order to get an estimate of ovaries volumes, the values obtained for the areas were transformed to radius

($r = \sqrt{\frac{A}{\pi}}$), thus approaching them as the areas of circles, and then these values were used to determine the volume of the ovaries by approaching them as the volumes of spheres ($\frac{4}{3}\pi r^3$).

Lifespan experiments

About 60 virgin females of each of the two strains (O53 (diapausing) and CB05.08 (non-diapausing)) were collected after eclosion, transferred to bottles containing standard medium and kept under diapause inducing conditions. After 28 days these female flies were individually transferred to single vials and then were reared in a climate chamber under non-diapause inducing conditions. At the same time 60 new born virgin females of each of both strains were collected and individually transferred to single vials containing standard medium and were introduced in the same climate chamber under non-diapause inducing conditions. All the vials were checked every other day and the individuals were changed into new vials every week until they were dead in order to determine their LS. Non-parametric tests, implemented in SPSS Statistics 22.0 (IBM, New York, USA), were used to test for significant differences between LS distributions.

Gene expression

The expression levels of *Actin79B* (known as *ActinD1* in *D. virilis*; see [42]) drop dramatically with adult age both in *D. melanogaster* and *D. virilis* (a species closely related to *D. americana*). Thus, in order to determine if the pattern of expression decay observed at 25°C is observed at 11°C and if it is different between diapausing and non-diapausing females, the *ActinD1* expression levels were determined in new born flies (collected at the same day of eclosion) and 28 days old virgin females raised under 12L:12D at 25°C and 10L:14D at 11°C. Three independent sets of two new born females, as well as three independent sets of two 28 days old virgin females in reproductive diapause (strain O53) and in non-reproductive diapause (strain CB05.08) were snap-frozen in liquid nitrogen. For controls, three independent sets with two 28 days old females showing fully mature ovaries were also snap-frozen. In order to determine if *ActinD1* expression levels could be used as a molecular marker of biological age (see Results), three independent sets of two 15 days old virgin O53 females and three independent sets of two 20 days old virgin CB05.08 females were also collected. All flies were stored at -80°C for further RNA extractions. Sets of two females were used for each condition to guarantee an RNA concentration high enough for further cDNA synthesis.

Independent pools containing nine females of strains from the north and pools containing nine females of strains from the south of the *D. americana* distribution reared under 12L:12D at 25°C, as well as under 10L:14D at 11°C were also prepared, in order to confirm that the expression levels obtained for *ActinD1* are not caused by the specific backgrounds of O53 and CB05.08. For each condition, three independent pools consisting of one female of each of the nine northern strains showing over 90% of diapause incidence (O31, O33, O37, O39, O43, O50, O53, O62, and O67; Table 1) and three independent pools containing one female of each of the nine strains from the south of the distribution showing less than 15% of diapause incidence (CB05.08, CB05.20, CB05.22, CB05.24, CI05.28, RB10.20, RB10.22, SF12, and SF14; Table 1) were snap frozen and stored at -80°C for further mRNA extraction.

Total RNA was isolated from whole bodies of virgin females of each sample using TRIzol Reagent (Invitrogen, Spain) according to the manufacturer's instructions and treated with Turbo DNA-free kit (Life technologies, Carlsbad, California, USA). The purity and concentration of each sample extracted was measured with NanoDrop ND-1000 spectrophotometer (NanoDrop, Thermo Scientific, Portugal) and RNA integrity was checked using Experion platform (Bio-Rad, Portugal; all the samples had RQI values above 8.6). cDNA was synthesized by

Table 1. Reproductive diapause incidence on *D. americana* strains from north, center and south of the distribution.

Strain	Diapause	Non-Diapause	% diapause
	11°C; 14D:10L; 28 days		
O31	20	0	100
O33	20	0	100
O37	15	0	100
O40	20	0	100
O43	20	0	100
O53	20	0	100
O67	19	1	95
O39	18	2	90
O50	18	2	90
O62	18	2	90
O30	16	4	80
O34	16	4	80
O57	16	4	80
O35	11	9	55
HI14	4	9	31
HI18	10	10	50
HI23	9	12	43
HI25	4	15	21
HI29	9	1	90
W11	15	3	83
W25	3	17	15
W26	13	0	100
W29	0	20	0
W37	16	4	80
W46	15	5	75
CB05.08	0	20	0
CB05.20	2	11	15
CB05.22	0	19	0
CB05.24	0	20	0
RB10.20	1	17	6
RB10.22	1	12	8
CI05.20	7	13	35
CI05.28	2	18	10
CI05.30	0	13	0
SF12	0	20	0
SF14	0	20	0

doi:10.1371/journal.pone.0138758.t001

reverse transcription of 1.0 μg of RNA of each sample with SuperScript III First-Strand Synthesis SuperMix for qRT-PCR (Invitrogen, Spain) using random primers. Reactions where template was not added and reactions with RNA that was not reverse transcribed were performed to confirm the absence of genomic DNA contamination. qPCR was performed using two technical replicates of all the cDNA samples and specific primers for *ActinD1* and the reference gene *RpL32* with the iQ SYBR Green Supermix (Bio-Rad, Portugal) according to the manufacturer's instructions on a Bio-Rad iCycler with the following program: 3 min at 95°C; 40 cycles of 30 s at 94°C, 30 s at 56°C and 30 s at 72°C followed by a standard melt curve and 3 min at

95°C. Specific primers for *ActinD1* (F: 5' CTACTCGTTCACCACCAC 3' and R: 5' TACCGC CAGACAGCACAT 3') and for the reference gene *RpL32* (F: 5' ACAACAGAGTGCGTCGTC 3' and R: 5' ATCTCCTTGCGTTTCTTC 3') were developed based on the *D. americana* genomes [43]. We have confirmed that these primers have a unique pairing sequence using BLAST search and the qPCR reactions were followed by standard melt curves to confirm the presence of a single PCR product with the melting temperature similar to the expected sequences. The efficiencies of the primers were measured using serial dilutions of cDNA and they are in between 90 and 100%. Gene *RpL32* (also known as *rp49*) was used as reference gene, because it is commonly used in *D. melanogaster* (e.g. [37]), and although, it could be argued that the expression of this gene may be different between these two species, we have obtained similar CT values between treatments and controls. The means of normalized *ActinD1* expression were calculated using the $2^{-\Delta CT}$ method [44, 45] and the Student's *t* test was applied to the data to address if there were significant changes in gene expression.

Results

Reproductive diapause in *D. americana*

In *Drosophila*, reproductive diapause is considered if ovarian development in all oocytes is arrested prior to vitellogenesis (the most advanced oocyte is ≤ stage seven according to King [40]). In *D. americana*, the ovaries of non-diapausing flies reared under diapause inducing conditions for 28 days are vitellogenic, since they show a lot of chorionated oocytes. However, the area of these ovaries (1.37mm^2) corresponds to 57% of the area of the ovaries of flies reared for 28 days under non-diapausing conditions (2.41mm^2) (Fig 1), suggesting an abnormal or

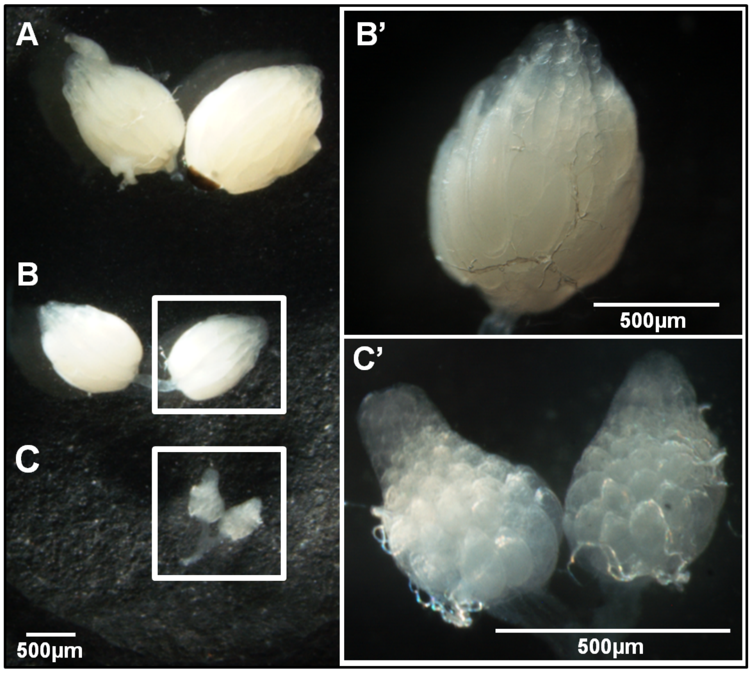

Fig 1. The phenotype of *D. americana* reproductive diapause. *Drosophila americana* 28 days-old ovaries of female flies reared since eclosion under non-diapausing conditions (12L:12D at 25°C, A), and reared since eclosion under diapause-inducing conditions (10L:14D at 11°C) showing non-diapause (B) or diapause (C) phenotypes. The non-diapausing (B') and diapausing ovaries (C') are also shown with higher magnifications. A scale of 500μm is provided in all pictures.

doi:10.1371/journal.pone.0138758.g001

delayed development caused by diapause inducing conditions alone. On the other hand, the ovaries of diapausing flies show previtellogenic oocytes only, and their area (0.26mm^2) is 19% of the ovaries area of flies reared under the same conditions, but that are unable to diapause (1.37 mm^2) (Fig 1). The area of these ovaries was used as a proxy to size, but these are tridimensional structures. When the values of the areas are transformed into volumes (see Materials and Methods for details), the size of ovaries of diapausing flies (0.10 mm^3) is now 8% of the size of ovaries of non-diapausing flies reared under diapause inducing conditions for 28 days (1.20mm^3), and non-diapausing ovaries show 43% of the size of the ovaries of flies reared for 28 days under non-diapausing conditions (2.81mm^3). Thus, the differences in ovarian sizes are even higher, when the volumes are considered. These results demonstrate that, after 28 days, *D. americana* diapausing flies show an explicit phenotype of underdeveloped ovaries which is clearly distinguishable from the non-diapausing phenotype.

Geographic variation in diapause incidence

The frequency of diapausing females, at 28 days of age raised since eclosion under diapause inducing conditions, was determined for 14 isofemale strains from the north, 11 from the centre and 11 from the south of the distribution (Table 1). As expected, in the northern population at least 55% of the individuals of each strain were in diapause and for 93% of the strains more than 80% of the females showed a diapausing phenotype. The strains from the center of the distribution (from two different populations) show a great variation in diapause incidence, from 0% to 100%, but for 36% of the strains more than 80% of the individuals showed a diapausing phenotype. A very low diapause incidence is observed for strains from the three southernmost populations ranging from 0% to 35% with more than half of the strains showing no diapausing individuals. The proportions of diapausing females are not different between the two populations from the center (Fisher's exact test, P = 0.110) nor between the three populations from the south (Fisher's exact test, P = 0.582, P = 0.115 and P = 0.729 for CB v. RB, CB v. CI&SF and RB v. CI&SF, respectively). Moreover, the proportion of diapausing females from the northern population of Omaha is significantly different from all proportions of diapausing females observed for the other five populations (Fisher's exact test; 1.70E-47< P < 6.90E-13) and both populations from the center show significant differences in the proportion of diapausing females when compared with the three southernmost populations (Fisher's exact test; 1.55E-15< P < 1.09E-4). When the data obtained for all the strains are grouped into northern, center and southern populations, the differences are even more significant (Fisher's exact test; P = 1.74E-21, P = 6.13E-82 and P = 1.98E-23 for north v. center, north v. south and center v. south, respectively). Therefore, in *D. americana*, as expected given the observations in other *Drosophila* species [6, 7, 9, 33], diapause incidence increases with latitude. When the percentages of diapausing females are plotted against latitude, almost 70% of the diapause incidence can be explained by the geographical origin of the different populations (Fig 2).

Diapausing flies live longer

Following the observation of negligible senescence of *D. melanogaster* diapausing females [15, 36], we determined the LS of *D. americana* diapausing and non-diapausing females and the LS of their respective controls. Since, there are significant differences on the average LS between *D. americana* from the north and south of the distribution [43], that if ignored could confuse the interpretation of the results, we first assayed the average LS of strain O53 (from the north; diapausing) and CB05.08 (from the south; non-diapausing). When raised under 12L:12D at 25°C, these two strains show no statistically significant difference regarding LS (Non-parametric Mann-Whitney Test; P > 0.05; see also Table 2), and thus, we can directly compare the

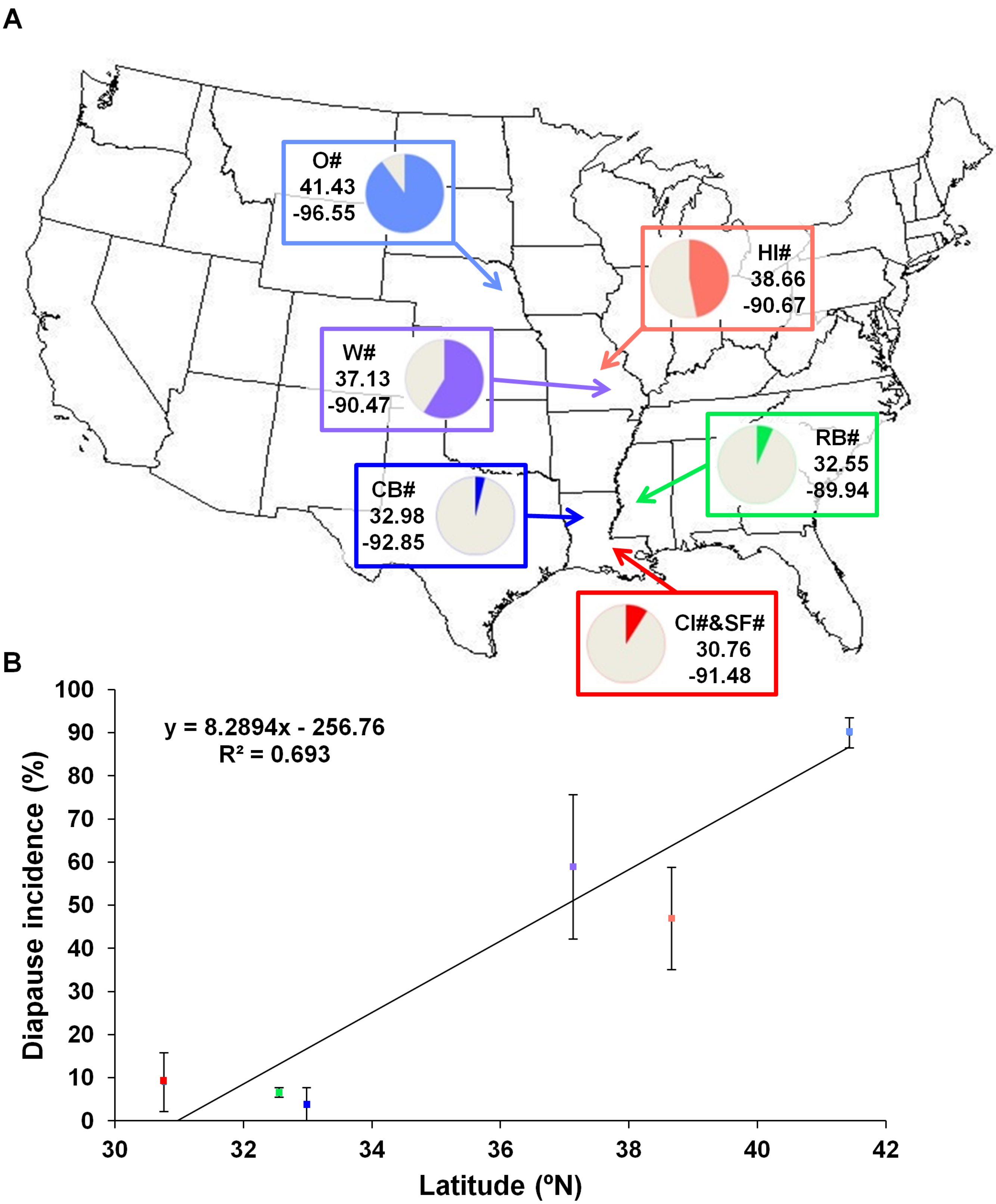

Fig 2. Geographical origin and diapause incidence of different populations of *D. americana* across the USA. Diapause frequencies are represented by the color portion of the circular graphics close to the geographical coordinates and collection sites (O–Fremont, Nebraska, HI–Howell Island, Missouri; W–Lake Wappapelo, Missouri; RB–Pearl River, Mississippi; CB–Corney Bayou, Louisiana; and CI&SF–Cat Island, Saint Francisville, Louisiana) of the strains

phenotyped (A). Diapause frequencies of each strain were used for the linear regression with the latitude of origin, and are presented by the average ± standard error of the mean (S.E.M) for each population. The slope of the curve represents the increase on diapause incidence with latitude and the R^2 value, the amount of variation on this phenotype explained by latitude (69.3%) (B).

doi:10.1371/journal.pone.0138758.g002

results obtained for the two strains in order to dissociate the effect of the diapause inducing conditions from the diapause effect *per se*.

When females are reared for 28 days under diapause inducing conditions and then transferred to non-diapause inducing conditions, they live significantly longer (15.9% for strain O53, and 10.0% for strain CB05.08) than their controls reared exclusively under non-diapause inducing conditions (Non-parametric Mann-Whitney Test; $P < 0.005$ and $P < 0.001$ for strains O53 and CB05.08, respectively; see also Table 2). Given that CB05.08 is a non-diapausing strain (Table 1), the LS extension observed for this strain is a direct consequence of the diapause inducing conditions *per se*. Under the assumption of a similar effect of the diapause inducing conditions *per se* on strain O53, diapause itself is responsible for the observed additional LS extension of 5.9%. The LS extension difference between the two strains is borderline non-significant (Non-parametric Mann-Whitney Test; $P = 0.055$).

Since there are no LS differences when comparing O53 and CB05.08 controls (see above), it seems reasonable to assume that the two strains have a similar death rate, and that the LS extension difference of 5.9% must have happened in the first 28 days. Under these assumptions we estimate a reduction in the ageing rate of 47.1% for diapausing and 28.6% for non-diapausing females when raised under diapause inducing conditions. Therefore, although there is an ageing slow down, senescence is far from being negligible and the biological ages estimated for O53 diapausing and CB05.08 non-diapausing females after 28 days are 14.2 and 20.0 days old, respectively. Moreover, when we measure LS starting from the day the flies were transferred into non-diapause inducing conditions, both CB05.08 and O53 females live 25.0% and 17.7% less than the controls, respectively (Non-parametric Mann-Whitney Test; $P < 0.001$ for both CB05.08 and O53) (Fig 3), showing again that senescence is far from being negligible during the period flies remained in diapause inducing conditions.

The *ActinD1* expression level is highly correlated with LS extension

Given that LS is extended both in diapausing and in non-diapausing females reared under diapause inducing conditions, we hypothesized that flies under those conditions should have a gene expression pattern that is compatible with flies younger than their chronological age. Both in *D. melanogaster* and *D. virilis* (a species closely related to *D. americana*), *Actin79B*

Table 2. Lifespan (in days) summary statistics for diapausing (O53-D) and non-diapausing (CB05.08-ND) females reared under diapause inducing conditions (10L:14D at 11°C) for 28 days and then transferred into non-diapause inducing conditions (12L:12D at 25°C) and controls (C) reared exclusively under non-diapause inducing conditions.

	N	Mean	St-dev	S.E.M	Range
O53-D (11°C+25°C)	58	96.4	23.7	3.1	42–134
O53-D (25°C)	58	68.4	23.7	3.1	14–106
CB05.08-ND (11°C+25°C)	58	88.0	23.5	3.1	41–129
CB05.08-ND (25°C)	58	60.0	23.5	3.1	13–101
O53-C	60	83.2	22.2	2.9	17–111
CB05.08-C	60	80.0	16.3	2.1	31–101

LS was determined since the day females were born (11°C+25°C and C) or since the day they were transferred into non-diapause inducing conditions (25°C)

doi:10.1371/journal.pone.0138758.t002

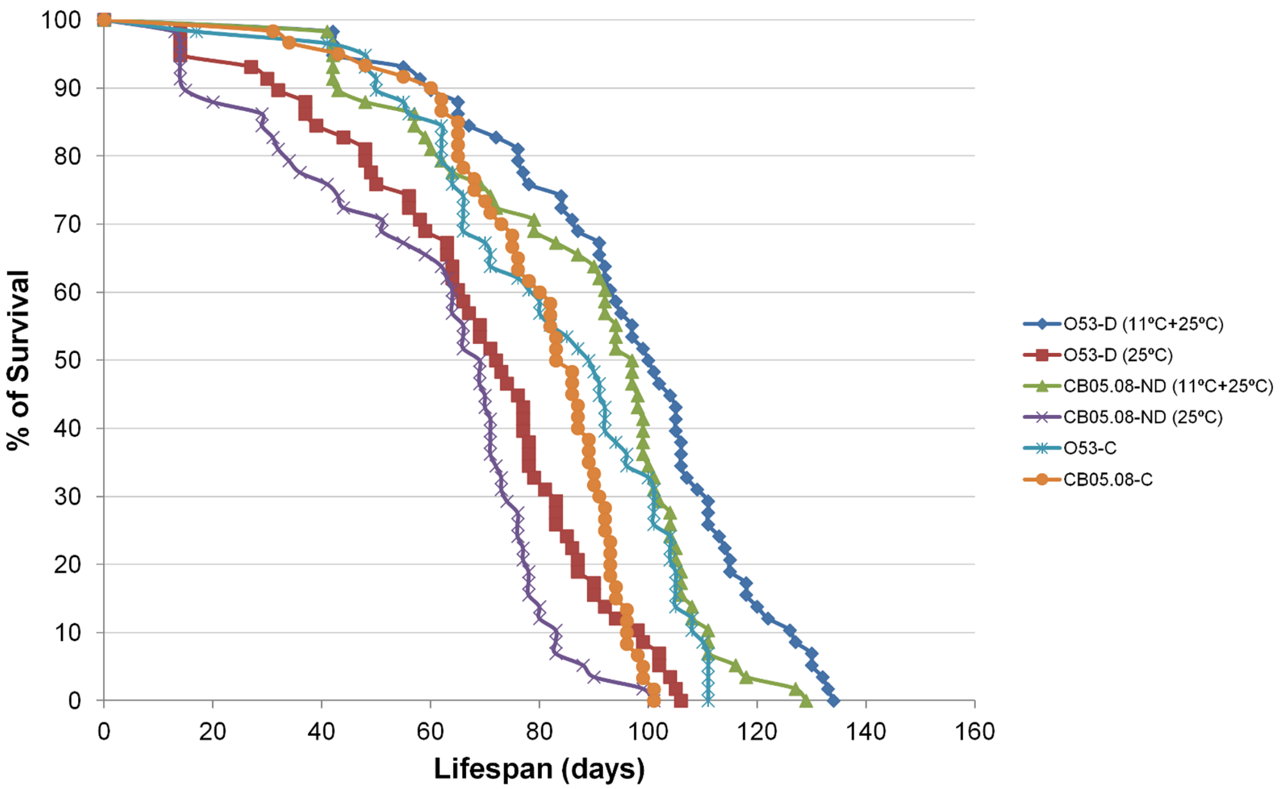

Fig 3. Diapause inducing conditions increase LS, which is more pronounced in diapausing females when compared with non-diapausing ones. Survival curves throughout time are shown for *D. americana* strains O53 (diapausing) and CB05.08 (non-diapausing) reared under non-diapause inducing conditions (12L:12D at 25°C; controls) and at the same conditions after being reared for 28 days under diapause inducing conditions (10L:14D at 11°C; O53-D (11°C+25°C) and CB05.08-ND (11°C+25°C)). Additional curves are presented for the same O53-D and CB05.08-ND females, but with LS measured starting from the day they were transferred into non-diapause inducing conditions (25°C).

doi:10.1371/journal.pone.0138758.g003

(known as *ActinD1* in *D. virilis*; see [42]) expression levels drop dramatically with adult age. Thus, we hypothesized that the expression of this gene could be a marker of biological age. This gene is expressed at high levels in the TDT (tergal depressor of the trochanter) muscle and in some other tubular muscles of the legs both in *D. melanogaster* [46] and in *D. virilis* [42]. Moreover, there is very little or even no expression in ovaries (modENCODE project ([47]; Flybase.org)). Therefore, *ActinD1* expression levels are independent of the oocyte stages which are used to characterize a female fly as diapausing or non-diapausing. Our results indicate that, as expected, in *D. americana*, *ActinD1* expression is highest in newborn females followed by diapausing individuals, non-diapausing individuals reared under diapause inducing conditions and finally in individuals reared under 12L:12D at 25°C (Fig 4). When *ActinD1* expression levels of females reared under diapause and non-diapause inducing conditions are plotted against the average LS determined in the previous section, 94% of the variation is explained ($R^2 = 0.94$; $P < 0.001$; Fig 5). Thus, also as expected, the *ActinD1* expression levels accurately reflect the observed differences in LS. According to the ageing slowdown rates estimated in the previous section, after 28 days under 10L:14D at 11°C, O53 diapausing and CB05.08 non-diapausing females should be of the same biological age as 15 and 20 days old females reared under 12L:12D at 25°C, respectively. Nevertheless, the expression levels of 15 days old O53 females and 20 days old CB05.08 females reared under non-diapause inducing conditions are significantly lower (Student's *t* test; $P < 0.01$) than those from females reared under diapause inducing conditions. They are, however, not statistically different from 28 days old controls (Fig 6).

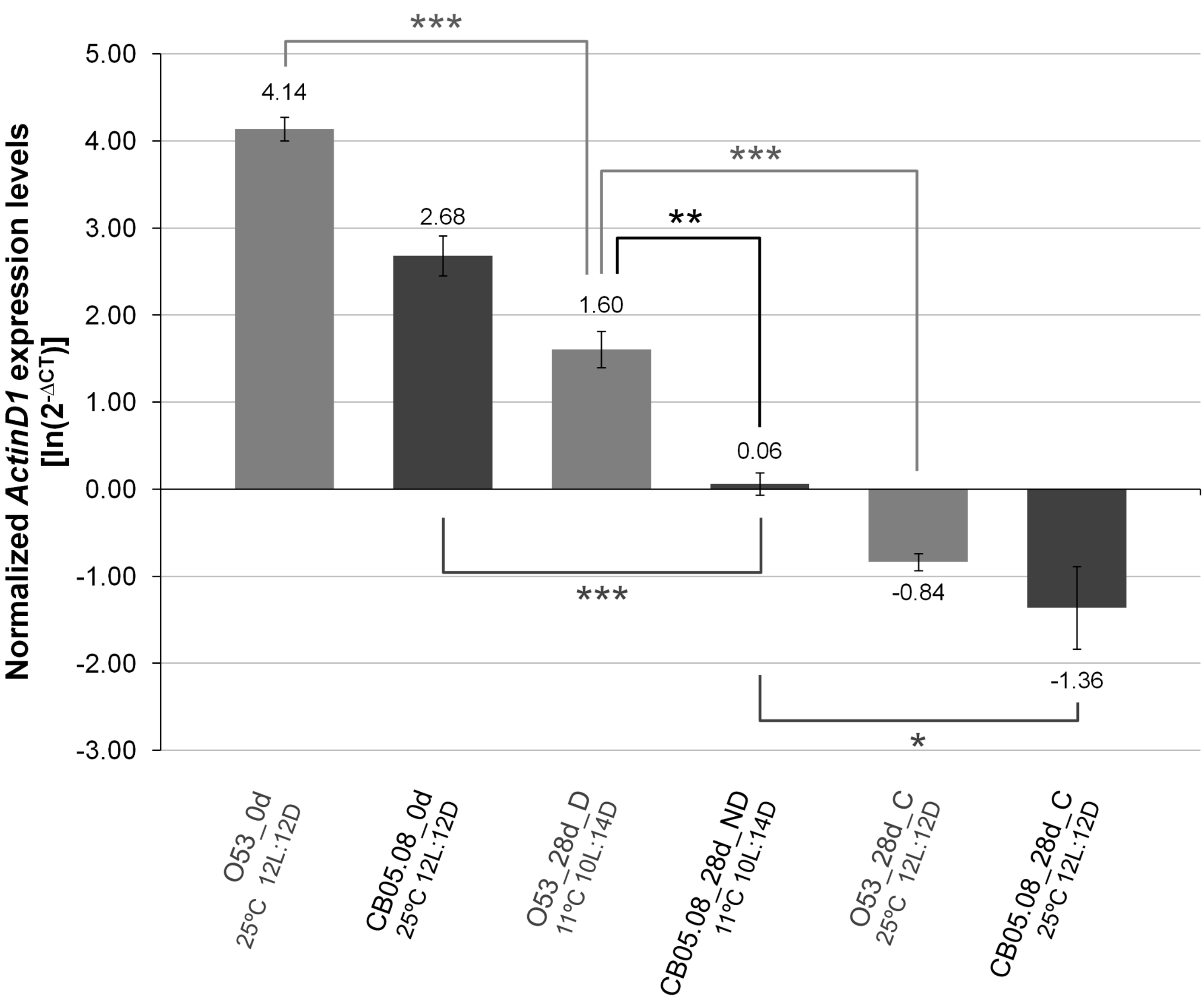

Fig 4. *ActinD1* expression levels drop slowly under diapause inducing conditions. Differences in *ActinD1* gene expression levels were addressed between new born females (0d), diapausing (D) and non-diapausing (ND) 28 days old females reared under diapause inducing conditions (10L:14D at 11°C) and controls (C) reared for 28 days under non-diapause inducing conditions (12L:12D at 25°C). The reference gene *RpL32* was used to normalize the expression values. Three biological replicates were used for each sample and the averages of the log-transformed values of $2^{-\Delta CT}$ are presented with their respective S.E.M. A two-tailed Student's *t* test assuming equal variances was used to address if the averages of normalized *ActinD1* expression levels of the different samples and treatments are significantly different (* 0.05 > P > 0.01; ** 0.01 > P > 0.001; *** P < 0.001).

doi:10.1371/journal.pone.0138758.g004

Thus, the slowing down in the rate of ontogenetic development induced by the diapause inducing conditions and diapause *per se* (reflected in the *ActinD1* expression levels), is greater than the slowdown in the ageing rate. Compatible with the ontogenetic delay caused by diapause inducing conditions, as well as by diapause *per se*, is the observation made in this work that, in *D. americana*, ovaries are smaller when both diapausing and non-diapausing flies are exposed to diapause inducing conditions, and that diapausing females have the smallest ovaries. Since, *ActinD1* shows very little or even no expression in ovaries, the observed ontogenetic delay must affect the fly as a whole.

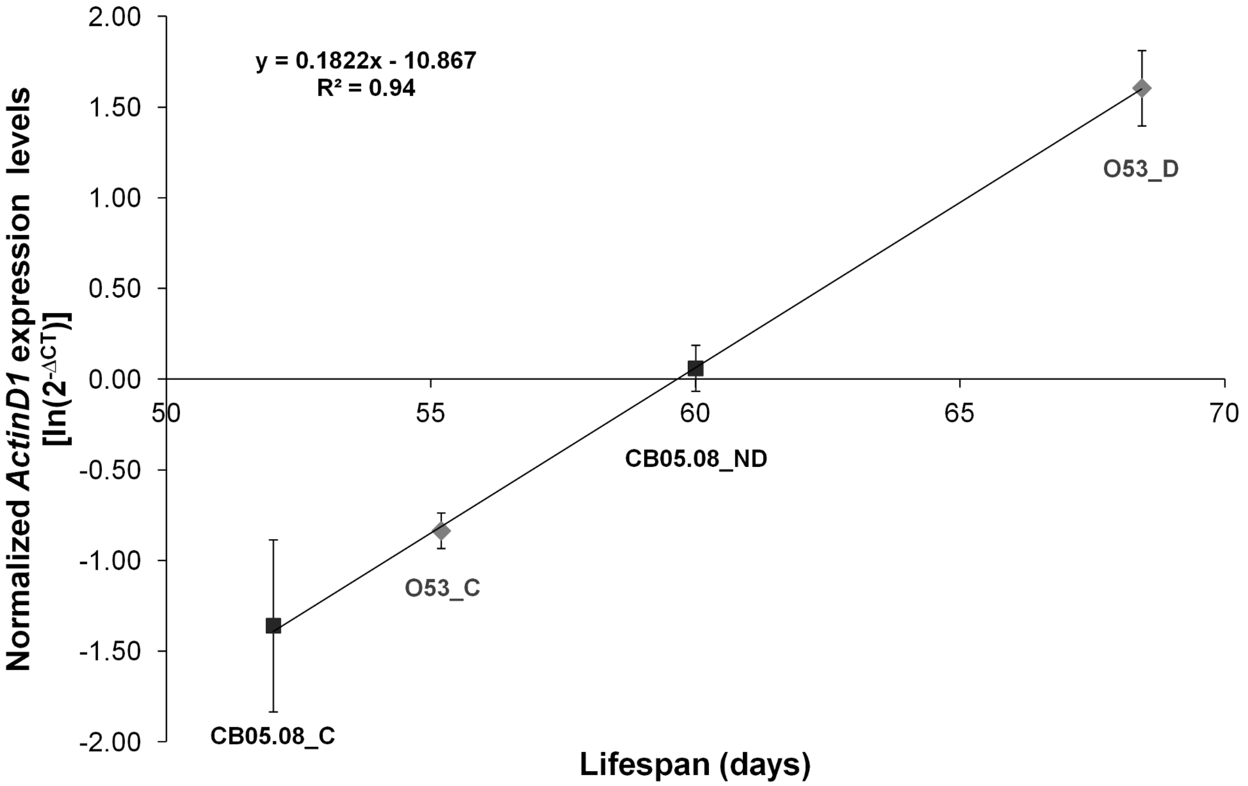

Fig 5. *ActinD1* expression levels are highly correlated with LS expectancy. Fold changes in *ActinD1* expression levels obtained for 28 days old controls (O53-C and CB05.08-C), as well as for 28 days old diapausing (O53-D) and non-diapausing females (CB05.08-ND) were plotted against their respective LS values obtained under 12L:12D at 25°C (excluding the first 28 days). Three biological replicates were used for each sample, and the individual log-transformed expression values were used for the linear regression. The averages of the log-transformed values of $2^{-\Delta CT}$ are presented with their respective S.E.M. The slope of the curve represents the increase in *ActinD1* expression levels with increased LS expectancy, and the R^2 value the amount of variation in expression levels explained by LS (94%).

doi:10.1371/journal.pone.0138758.g005

In order to address whether our conclusions could be affected by the use of a single diapausing and a single non-diapausing strain, qPCR was performed using pools containing females of different strains from the north showing over 90% of diapause incidence and pools containing females from different strains from the south showing less than 15% of diapause incidence, reared under 12L:12D at 25°C or 10L:14D at 11°C. The *ActinD1* expression levels obtained for pools of diapausing females are similar to those obtained for O53 diapausing females, and the expression levels of pools of non-diapausing females and CB05.08 non-diapausing females are also similar. Similar results were obtained when control pools from the north and south were compared with O53 and CB05.08 controls, respectively. It should be noted that when using these pools, the differences between diapausing and non-diapausing females remained significant (Fig 7).

Discussion

D. americana diapausing females show the typical features of diapause observed in other *Drosophila* species, such as small ovary size associated with underdeveloped oocytes and higher incidence with increased latitude [6, 7, 9, 33]. It should be noted that in this species, under the conditions used here (10L:14D at 11°C; the usual conditions for *D. melanogaster*; [14, 48]), non-diapausing females from the very south of the distribution take about three to four weeks

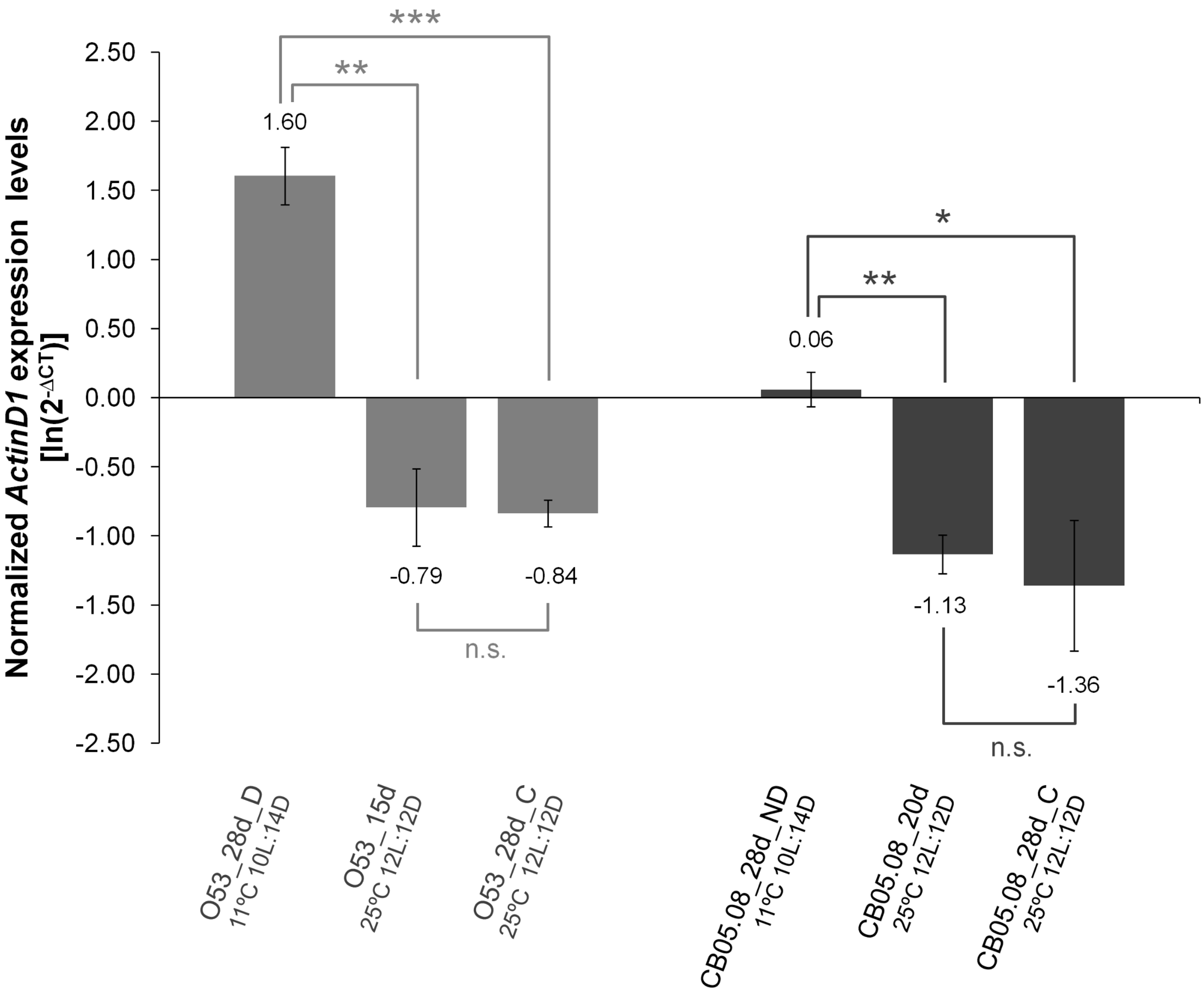

Fig 6. *ActinD1* expression level can be used as a marker of ontogenetic delay, which results in extended LS, but it is not a marker of biological age. Differences in *ActinD1* gene expression levels were determined between 28 days old diapausing females (O53-D), 15 days old (O53-15d) and control females (O53-C) reared for 28 days under non-diapause inducing conditions (12L:12D at 25°C), as well as between 28 days old non-diapausing females (CB05.08-ND), 20 days old (CB05.08-20d) and controls (CB05.08-C) reared for 28 days under non-diapause inducing conditions (12L:12D at 25°C). The reference gene *RpL32* was used to normalize the expression values. Three biological replicates were used for each sample and the averages of the log-transformed values of $2^{-\Delta CT}$ are presented with their respective S.E.M. A two-tailed Student's *t* test assuming equal variances was used to address if the averages of normalized *ActinD1* expression levels of the different samples and treatments are significantly different (n.s. P > 0.05; * 0.05 > P > 0.01; ** 0.01 > P > 0.001; *** P < 0.001).

doi:10.1371/journal.pone.0138758.g006

to develop vitellogenic ovaries (data not shown). Therefore, in order to clearly distinguish the two phenotypes, it is best to look at four week old females.

An increase in LS was observed for non-diapausing females reared under diapause inducing conditions. This is not surprising, since it is well established that LS is extended at low temperatures in the absence of diapause [43, 49, 50]. For instance, in *D. americana*, a decrease of 7°C (from 25°C to 18°C), on average, extends by two and three times the LS of females and males, respectively [43]. This observation is compatible with that obtained for *D. melanogaster*

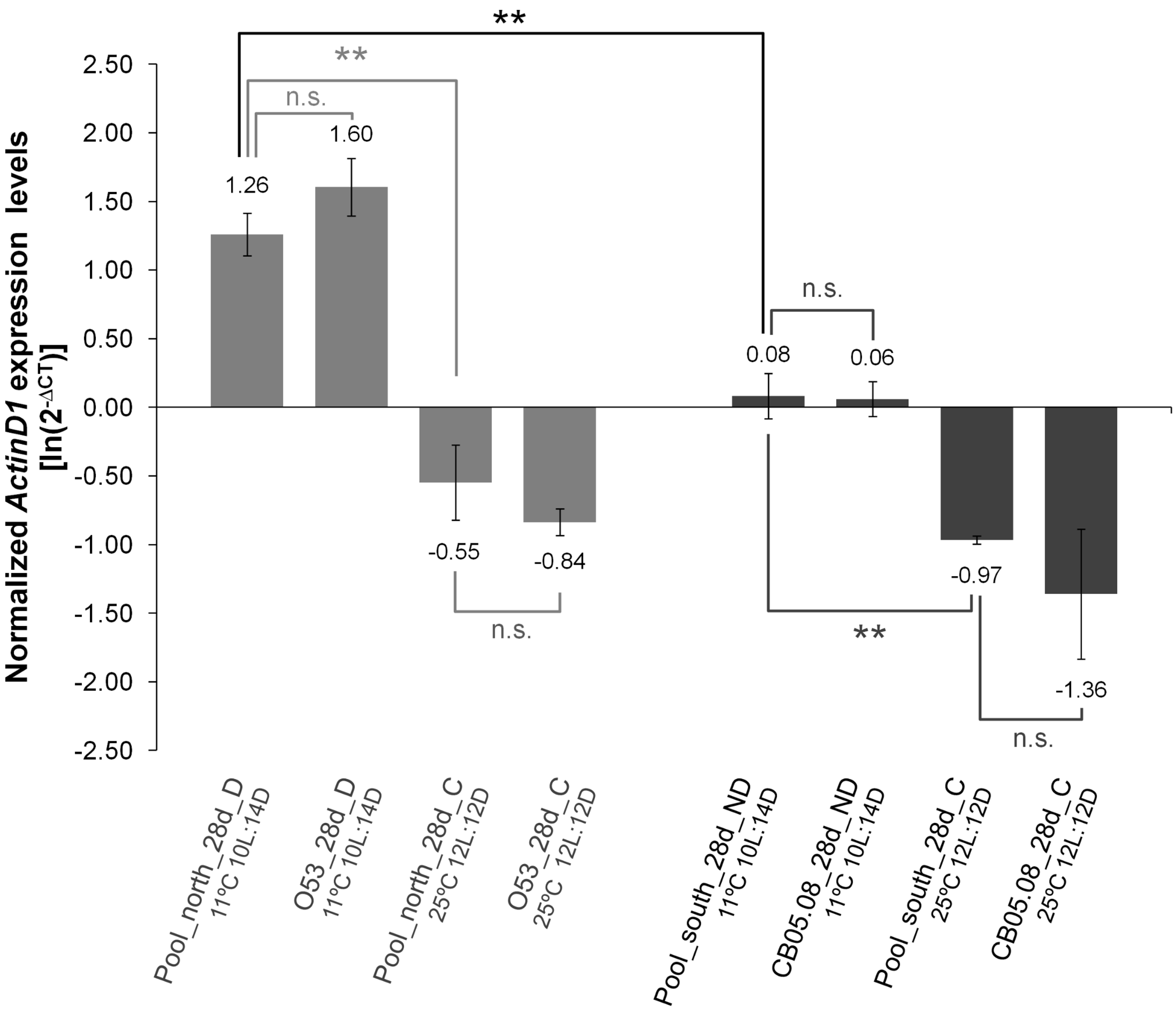

Fig 7. The pattern of *ActinD1* expression is not specific of the genetic background of the two strains used in this work. *ActinD1* gene expression levels of pools of 28 days old diapausing females from nine strains from the north of the distribution showing over 90% of diapause incidence and pools of 28 days old control flies reared under non-diapause inducing conditions were compared with the expression levels obtained for O53 diapausing females and controls. The expression levels of *ActinD1* were also obtained for pools of 28 days old non-diapausing females from nine strains from the south of the distribution showing less than 15% of diapause incidence and pools of 28 days old control flies reared under non-diapause inducing conditions and then they were compared with the expression levels obtained for CB05.08 non-diapausing females and controls. The reference gene *RpL32* was used to normalize the expression values. Three biological replicates were used for each sample and the averages of the log-transformed values of $2^{-\Delta CT}$ are presented with their respective S.E.M. A two-tailed Student's *t* test assuming equal variances was used to address if the averages of normalized *ActinD1* expression levels of the different samples and treatments are significantly different (n.s. P > 0.05; ** 0.01 > P > 0.001).

doi:10.1371/journal.pone.0138758.g007

[49, 50]. In the northern *D. americana* strain, however, LS extension is not due to exposure to diapause-inducing conditions alone. Indeed, non-diapausing females reared for 28 days under these conditions and then at 25°C, do not show a LS extension as large as diapausing females reared under the same conditions. By comparing these two observations we can rule out the effect of temperature/photoperiod alone and estimate that diapause itself extends LS by about

18.6%. Nevertheless, when we start counting longevity from the moment diapausing females are transferred to the same conditions as controls, (under 12L:12D at 25°C after being reared at 11°C under 10L:14D for 28 days) they do not live as long as controls (raised exclusively at 25°C under 12L:12D). Thus, senescence in virgin diapausing females is far from being negligible. This is in sharp contrast to what has been reported for *D. melanogaster* [15, 36]. Other *Drosophila* species must be analyzed as well, in order to determine whether, in general, senescence is negligible in diapausing females.

In *D. melanogaster* and *D. virilis* (a species closely related to *D. americana*) adults, the *ActinD1* expression level drops dramatically with age [42]. The *ActinD1* expression levels of 28 days old *D. americana* non-diapausing females reared under diapause inducing conditions suggest that they are younger than their chronological age, and that diapausing females are even biologically younger than non-diapausing ones reared under the same conditions. The pattern of expression of *Culex pipiens Actin 2* gene (the ortholog of *D. americana ActinD1*) in diapausing mosquitoes (high expression levels early in diapause that decay with time [51]) is compatible with this view. It should be noted that in *Drosophila* adults, this gene is expressed at high levels in the TDT muscle and in some other tubular muscles of the legs [42, 46], and that there is very little or no expression in ovaries (modENCODE project ([47]; Flybase.org)). The slowdown in the rate of ontogenetic development induced by the diapause inducing conditions and diapause *per se* (reflected in the *ActinD1* expression levels) is, however, greater than the slowdown in the ageing rate. It has been suggested that the slowing down of the ageing rate could be a consequence of the overall depression of the fly metabolism due to the diapausing state [37], and the same could be true for the rate of ontogenetic development. Our observations suggest that the development of the whole fly, rather than the ovarian development alone (which is phenotypically more evident [6–10]) is delayed by the diapause inducing conditions and by diapause *per se*. Compatible with this view, is the observation that the rate of senescence of *D. melanogaster* female intestines is slowing down under diapause inducing conditions [37].

Kankare *et al.* [52] reported significant differences in expression levels for 24 out of 101 genes when comparing young (one day old), diapausing (14 days old) and reproducing (14 days old) *D. montana* females. Not surprisingly, 16 out of these 24 genes show significant changes when comparing reproducing versus young flies. The same patterns are observed for these 16 genes in *D. melanogaster* according to the modENCODE project ([47]; Flybase.org), revealing significant ageing in reproducing flies. On the other hand, only seven out of the 24 genes show significant differences between diapausing and young females. Thus, according to gene expression, diapausing females are biologically older than young (one day old) flies, but younger than reproducing ones of the same chronological age. Therefore, for some genes, diapausing females show expression levels compatible with flies younger than their chronological age, making it unclear whether such changes could be solely due to the ageing slowdown that seems to be typical of diapause. The identification of genes that are specifically up and down regulated due to the diapause state itself may be thus, more difficult than anticipated.

In summary, in *D. americana*, diapause is a genetically determined delay in ovarian development that is triggered by temperature and/or photoperiod. Flies that are reared under diapause inducing conditions are biologically younger than their chronological age, and show a delay in the rate of ontogenetic development, and this effect is further exacerbated in diapausing females. Nevertheless, in *D. americana* diapausing females, under the conditions used here, senescence is far from being negligible.

Author Contributions

Conceived and designed the experiments: MR CPV JV. Performed the experiments: MR FBV CPV JV. Analyzed the data: MR FBV CPV JV. Wrote the paper: MR FBV CPV JV.

References

1. Henneguy LF (1904) Les insectes: morphologie, reproduction, embryogénie: Masson, Paris.
2. Hahn DA, Denlinger DL (2011) Energetics of insect diapause. Annu Rev Entomol 56: 103–121. doi: 10.1146/annurev-ento-112408-085436 PMID: 20690828
3. Danks HV (1987) Insect dormancy: an ecological perspective. Ottawa: Biological Survey of Canada (Terrestrial Artropods).
4. Bale JS, Hayward SAL (2010) Insect overwintering in a changing climate. J Exp Biol 213: 980–994. doi: 10.1242/jeb.037911 PMID: 20190123
5. Tauber MJ, Tauber CA, Masaki S (1986) Seasonal adaptations of insects. New York: Oxford University Press.
6. Lankinen P (1986) Genetic correlation between circadian eclosion rhythm and photoperiodic diapause in *Drosophila littoralis*. J Biol Rhythms 1: 101–118. PMID: 2979577
7. Ichijo N (1986) Disjunctive cline of critical photoperiod in the reproductive diapause of *Drosophila lacertosa*. Evolution 40: 418–421.
8. Schmidt PS, Paaby AB (2008) Reproductive diapause and life-history clines in North American populations of *Drosophila melanogaster*. Evolution 62: 1204–1215. doi: 10.1111/j.1558-5646.2008.00351.x PMID: 18298646
9. Tyukmaeva VI, Salminen TS, Kankare M, Knott KE, Hoikkala A (2011) Adaptation to a seasonally varying environment: a strong latitudinal cline in reproductive diapause combined with high gene flow in *Drosophila montana*. Ecology and Evolution 1: 160–168. doi: 10.1002/ece3.14 PMID: 22393492
10. Yamada H, Yamamoto MT (2011) Association between circadian clock genes and diapause incidence in *Drosophila triauraria*. PLoS ONE 6: e27493. doi: 10.1371/journal.pone.0027493 PMID: 22164210
11. Lumme J (1978) Phenology and photoperiodic diapause in northern populations of *Drosophila*. Evolution of insect migration and diapause: Springer. pp. 145–170.
12. Watabe H (1983) Photoperiodic responses in the *Drosophila virilis* species group (Diptera, Drosophilidae) from Japan. Jap J Entomol 51: 628–634.
13. Kimura MT (1984) Geographic variation of reproductive diapause in the *Drosophila auraria* complex (Diptera: Drosophilidae). Physiol Entomol 9: 425–431.
14. Saunders DS, Henrich VC, Gilbert LI (1989) Induction of diapause in *Drosophila melanogaster*: photoperiodic regulation and the impact of arrhythmic clock mutations on time measurement. Proc Natl Acad Sci U S A 86: 3748–3752. PMID: 2498875
15. Tatar M, Chien SA, Priest NK (2001) Negligible senescence during reproductive dormancy in *Drosophila melanogaster*. Am Nat 158: 248–258. doi: 10.1086/321320 PMID: 18707322
16. Salminen TS, Hoikkala A (2013) Effect of temperature on the duration of sensitive period and on the number of photoperiodic cycles required for the induction of reproductive diapause in *Drosophila montana*. J Insect Physiol 59: 450–457. doi: 10.1016/j.jinsphys.2013.02.005 PMID: 23428942
17. Lankinen P, Tyukmaeva VI, Hoikkala A (2013) Northern *Drosophila montana* flies show variation both within and between cline populations in the critical day length evoking reproductive diapause. J Insect Physiol 59: 745–751. doi: 10.1016/j.jinsphys.2013.05.006 PMID: 23702203
18. Cogni R, Kuczynski C, Koury S, Lavington E, Behrman EL, O'Brien KR, et al. (2013) The intensity of selection acting on the *couch potato* gene—spatial-temporal variation in a diapause cline. Evolution.
19. Schmidt PS, Conde DR (2006) Environmental heterogeneity and the maintenance of genetic variation for reproductive diapause in *Drosophila melanogaster*. Evolution 60: 1602–1611. PMID: 17017061
20. Salminen TS, Vesala L, Hoikkala A (2012) Photoperiodic regulation of life-history traits before and after eclosion: egg-to-adult development time, juvenile body mass and reproductive diapause in *Drosophila montana*. J Insect Physiol 58: 1541–1547. doi: 10.1016/j.jinsphys.2012.09.007 PMID: 23026647
21. Vesala L, Salminen TS, Kankare M, Hoikkala A (2012) Photoperiodic regulation of cold tolerance and expression levels of regucalcin gene in *Drosophila montana*. J Insect Physiol 58: 704–709. doi: 10.1016/j.jinsphys.2012.02.004 PMID: 22360999
22. Sim C, Denlinger DL (2013) Insulin signaling and the regulation of insect diapause. Front Physiol 4: 189. doi: 10.3389/fphys.2013.00189 PMID: 23885240

23. Kauranen H, Tyukmaeva V, Hoikkala A (2013) Involvement of circadian oscillation(s) in the photoperiodic time measurement and the induction of reproductive diapause in a northern *Drosophila* species. J Insect Physiol 59: 662–666. doi: 10.1016/j.jinsphys.2013.04.007 PMID: 23665332
24. Allen M (2007) What makes a fly enter diapause? Fly 1: 307–310. PMID: 18820432
25. Williams KD, Busto M, Suster ML, So AK-C, Ben-Shahar Y, Leevers SJ, et al. (2006) Natural variation in *Drosophila melanogaster* diapause due to the insulin-regulated PI3-kinase. Proc Natl Acad Sci U S A 103: 15911–15915. PMID: 17043223
26. Flatt T, Tu M-P, Tatar M (2005) Hormonal pleiotropy and the juvenile hormone regulation of *Drosophila* development and life history. Bioessays 27: 999–1010. PMID: 16163709
27. Tauber E, Zordan M, Sandrelli F, Pegoraro M, Osterwalder N, Breda C, et al. (2007) Natural selection favors a newly derived timeless allele in *Drosophila melanogaster*. Science 316: 1895–1898. PMID: 17600215
28. Sandrelli F, Tauber E, Pegoraro M, Mazzotta G, Cisotto P, Landskron J, et al. (2007) A molecular basis for natural selection at the timeless locus in *Drosophila melanogaster*. Science 316: 1898–1900. PMID: 17600216
29. Schmidt PS, Zhu C-T, Das J, Batavia M, Yang L, Eanes WF (2008) An amino acid polymorphism in the *couch potato* gene forms the basis for climatic adaptation in *Drosophila melanogaster*. Proc Natl Acad Sci U S A 105: 16207–16211. doi: 10.1073/pnas.0805485105 PMID: 18852464
30. Lee SF, SgrÒ CM, Shirriffs J, Wee CW, Rako L, Van Heerwaarden B, et al. (2011) Polymorphism in the *couch potato* gene clines in eastern Australia but is not associated with ovarian dormancy in *Drosophila melanogaster*. Mol Ecol 20: 2973–2984. doi: 10.1111/j.1365-294X.2011.05155.x PMID: 21689187
31. Danks HV (2002) The range of insect dormancy responses. Eur J Entomol 99: 127–142.
32. Goto SG, Yoshida KM, Kimura MT (1998) Accumulation of *Hsp70* mRNA under environmental stresses in diapausing and nondiapausing adults of *Drosophila triauraria*. J Insect Physiol 44: 1009–1015. PMID: 12770438
33. Schmidt PS, Matzkin L, Ippolito M, Eanes WF (2005) Geographic variation in diapause incidence, life-history traits, and climatic adaptation in *Drosophila melanogaster*. Evolution 59: 1721–1732. PMID: 16331839
34. Schmidt PS, Paaby AB, Heschel MS (2005) Genetic Variance for Diapause Expression and Associated Life Histories in *Drosophila melanogaster*. Evolution 59: 2616–2625. PMID: 16526509
35. Vesala L, Hoikkala A (2011) Effects of photoperiodically induced reproductive diapause and cold hardening on the cold tolerance of *Drosophila montana*. J Insect Physiol 57: 46–51. doi: 10.1016/j.jinsphys.2010.09.007 PMID: 20932841
36. Tatar M, Yin CM (2001) Slow aging during insect reproductive diapause: why butterflies, grasshoppers and flies are like worms. Experimental Gerontology 36: 723–738. PMID: 11295511
37. Kubrak OI, Kucerova L, Theopold U, Nassel DR (2014) The sleeping beauty: how reproductive diapause affects hormone signaling, metabolism, immune response and somatic maintenance in *Drosophila melanogaster*. PLoS ONE 9: e113051. doi: 10.1371/journal.pone.0113051 PMID: 25393614
38. Throckmorton LH (1982) The *virilis* species group. In: Ashburner M, Novistky E, editors. The genetics and biology of *Drosophila*. London: Academic. pp. 227–297.
39. Morales-Hojas R, Reis M, Vieira CP, Vieira J (2011) Resolving the phylogenetic relationships and evolutionary history of the *Drosophila virilis* group using multilocus data. Molecular Phylogenetics and Evolution 60: 249–258. doi: 10.1016/j.ympev.2011.04.022 PMID: 21571080
40. King RC (1970) The meiotic behavior of the *Drosophila* oocyte. Int Rev Cytol 28: 125–168. PMID: 4908567
41. Schneider CA, Rasband WS, Eliceiri KW (2012) NIH Image to ImageJ: 25 years of image analysis. Nature methods 9: 671–675. PMID: 22930834
42. Lovato TL, Meadows SM, Baker PW, Sparrow JC, Cripps RM (2001) Characterization of muscle actin genes in *Drosophila virilis* reveals significant molecular complexity in skeletal muscle types. Insect Mol Biol 10: 333–340. PMID: 11520356
43. Fonseca NA, Morales-Hojas R, Reis M, Rocha H, Vieira CP, Nolte V, et al. (2013) *Drosophila americana* as a model species for comparative studies on the molecular basis of phenotypic variation. Genome Biol Evol 5: 661–679. doi: 10.1093/gbe/evt037 PMID: 23493635
44. Livak KJ, Schmittgen TD (2001) Analysis of relative gene expression data using real-time quantitative PCR and the $2^{-\Delta\Delta CT}$ method. Methods 25: 402–408. PMID: 11846609
45. Schmittgen TD, Livak KJ (2008) Analyzing real-time PCR data by the comparative C(T) method. Nature protocols 3: 1101–1108. PMID: 18546601

46. Courchesne-Smith CL, Tobin SL (1989) Tissue-specific expression of the *79B actin* gene during *Drosophila* development. Dev Biol 133: 313–321. PMID: 2499492

47. Graveley BR, Brooks AN, Carlson J, Duff MO, Landolin JM, Yang L, et al. (2011) The developmental transcriptome of *Drosophila melanogaste*r. Nature 471: 473–479. doi: 10.1038/nature09715 PMID: 21179090

48. Williams KD, Sokolowski MB (1993) Diapause in *Drosophila melanogaster* females: a genetic analysis. Heredity 71 (Pt 3): 312–317. PMID: 8407357

49. Miquel J, Lundgren PR, Bensch KG, Atlan H (1976) Effects of temperature on the life span, vitality and fine structure of *Drosophila melanogaster*. Mechanisms of Ageing and Development 5: 347–370. PMID: 823384

50. Loeb J, Northrop JH (1917) On the influence of food and temperature upon the duration of life. J Biol Chem 32: 103–121.

51. Kim M, Robich RM, Rinehart JP, Denlinger DL (2006) Upregulation of two actin genes and redistribution of actin during diapause and cold stress in the northern house mosquito, *Culex pipiens*. J Insect Physiol 52: 1226–1233. PMID: 17078965

52. Kankare M, Salminen T, Laiho A, Vesala L, Hoikkala A (2010) Changes in gene expression linked with adult reproductive diapause in a northern malt fly species: a candidate gene microarray study. BMC Ecol 10: 3. doi: 10.1186/1472-6785-10-3 PMID: 20122138
